# Luminescent/Temperature-Sensing Properties of Multifunctional Rare-Earth Upconversion Kevlar Nanofiber Composite under 1550 nm

**DOI:** 10.3390/nano14090740

**Published:** 2024-04-24

**Authors:** Juan Li, Shengang Xu, Yingliang Liu, Shaokui Cao

**Affiliations:** 1 School of Materials Science and Engineering, Zhengzhou University, Zhengzhou 450001, China; lijuan@hnas.ac.cn (J.L.); xusg@zzu.edu.cn (S.X.); 2 Henan Key Laboratory of Advanced Nylon Materials and Application, Zhengzhou University, Zhengzhou 450001, China; 3 Engineering Department, Huanghe Science and Technology College, Zhengzhou 450063, China

**Keywords:** rare-earth upconversion, near-infrared, Kevlar nanofiber, temperature sensing

## Abstract

The unique properties of upconversion nanoparticles (UCNPs) are responsible for their diverse applications in photonic materials, medicine, analytics, and energy conversion. In this study, water-soluble rare-earth upconversion nanomaterials emitting green, yellow, and red light under 1550 nm excitation were synthesized. These nanomaterials were then integrated into water-soluble Kevlar nanofibers (KNFs) to fabricate ultra-thin composite films exhibiting favorable mechanical characteristics. The characterization of the products, along with their luminescent, mechanical, and temperature-sensing properties, was examined. The results indicate that the composite material exhibited varying colors based on the doped nanoparticles when subjected to 1550 nm excitation. The composite showed highly sensitive temperature-sensing properties, excellent luminescent characteristics, and superior mechanical strength. This study suggests that KNFs are effective carriers of UCNPs. This study offers a reference for the utilization of rare-earth upconversion in anti-counterfeiting displays, wearable health monitoring, and remote temperature sensing.

## 1. Introduction

Upconversion luminescence refers to a phenomenon in which a short-wavelength, high-energy photon is emitted following the continuous absorption of two or more long-wavelength, low-energy photons [1]. Rare-earth-doped upconversion nanoparticles (UCNPs) exhibit different features such as sharp emission peaks, a broad spectral range, wide emission spectrum coverage, excellent photochemical stability, and prolonged fluorescence lifetime when excited by long wavelengths [2]. It has been reported that the material can find extensive applications in various fields such as fluorescent probes [3], temperature sensing [4], anti-counterfeiting display [5], solar cells [6], photocatalysis [7], biomedicine [8], and photoisomerization [9]. Temperature sensors utilizing UCNPs do not necessitate direct physical contact with the detected system and can accurately assess temperature within a limited range [10,11]. NaREF_4_ is acknowledged for its high-quality matrix properties attributed to its low phonon energy and excellent chemical stability [12]. Lanthanide ions such as Tm^3+^ and Er^3+^ exhibit a well-structured energy level configuration, enabling the emission of upconversion spectra across various spectral bands upon exposure to monochromatic near-infrared light [13]. Typically, Yb^3+^ ions are incorporated into these nanoparticles as sensitizer ions to facilitate energy transfer to activator ions (Er^3+^, Tm^3+^) under excitation at ~980 nm, thus enabling efficient upconversion [14,15]. Nanocrystals of NaYF_4_: Yb, Er are now considered to be among the most popular UCNPs. The efficient conversion of telecom wavelength (1.3–1.55 μm) into visible or NIR light is of particular importance due to their potential uses in frequency upconversion lasing [16], photovoltaics [6], the detection of surface plasmons [17], and telecommunications [18]. Er^3+^ ions are well suited for these endeavors owing to their absorption at ~1550 nm, which corresponds to the ^4^I_13/2_ energy level. The efficiency of upconversion is significantly influenced by the concentration of dopants [15].

Kevlar fiber, developed by DuPont, is a high-performance aromatic polyamide organic fiber. This material has found extensive applications in the aerospace, military, mechanical, and chemical industries due to its high strength, low density, excellent high-thermal stability, and resistance to chemical corrosion [19,20]. Kotov et al. [21] were the first to synthesize Kevlar nanofibers (KNFs). Nano-scale Kevlar fibers were discovered to preserve the exceptional characteristics of macro-scale Kevlar fibers while demonstrating favorable mechanical and thermal stability. With the advancement of KNF research, KNF composites have garnered significant attention in various applications such as pressure sensing [22], smart wearable fabrics [23], thermal management [24], super hydrophobic wear-resistant coating materials [25], impact resistance, and deformability of reinforcing materials [26].

The existing research concerning KNF composite materials predominantly concentrates on their mechanical properties, thermal stability, and flexibility. However, there is a lack of comprehensive studies on the mechanical properties, luminescent properties, and temperature-sensing properties of KNFs modified by UCNPs. The utilization of KNFs for temperature-sensing and anti-counterfeiting display applications represents a novel concept introduced for the first time in this study. Based on the water-soluble modification reaction of UCNPs and KNFs, composite films of UCNPs/KNFs were synthesized. Subsequently, an analysis was conducted on the luminescence, mechanical properties, and temperature-sensing properties of these composite films. As anticipated, this study successfully synthesized multi-functional composite materials that combine the luminescent and temperature-sensing characteristics of UCNPs with the high-strength properties of KNFs.

## 2. Materials and Methods

### 2.1. Materials

Oleic acid (OA, 90%), 1-octadecene (ODE, 90%), ammonium fluoride (NH_4_F, ≥99.99%), sodium acetate (NaOA, 98%), cyclohexane (C_6_H_12_, AR), potassium hydroxide (KOH, AR), epichlorohydrin (ECH, AR), dimethyl sulfoxide (DMSO, AR), yttrium chloride (III) hexahydrate (YCl_3_·6H_2_O, 99.99%), and erbium chloride (III) hexahydrate (ErCl_3_·6H_2_O, 99.99%) were procured from Shanghai Maclean Biochemical Technology Co., Ltd., Shanghai, China. Ethanol (CH_3_CH_2_OH, AR) was obtained from Tianjin Kemiou Chemical Reagent Co., Ltd., Tianjin, China. Polyethylene glycol (PEG 400, AR) was sourced from Shanghai Aladdin Biochemical Technology Co., Ltd., Shanghai, China. All these reagents were utilized without further purification. All the remaining solvents were procured from Sigma-Aldrich (St. Louis, MO, USA) and utilized without further purification. Kevlar was acquired from Henan Shenma Aramid Technology Development Co., Ltd., Pingdingshan, China.

### 2.2. Instruments and Characterization

The upconversion luminescence spectra were acquired using a PTI-QM40-Laser-NIR fluorescence spectrometer (Photon Technology International, Inc., Birmingham, NJ, USA) with excitation from a laser (1550 nm, LEO Photonics Co., Ltd., Shenzhen, China). The morphology of UCNPs and KNFs was analyzed using a JEOL JEM−2010F transmission electron microscope (TEM) (JEOL, Tokyo, Japan). The samples were prepared by depositing a diluted solution onto a micro-grid copper mesh and subsequently drying in air. The nanoparticle size distribution of UCNPs was analyzed using Nano Measure 1.2 software and was statistically evaluated across a sample of 300 nanoparticles. X-ray diffraction (XRD) analyses were conducted utilizing a D/max 2500 XRD diffractometer (Rigaku, Tokyo, Japan) with Cu Kα radiation (60 kV, 80 mA) at a scanning rate of 10°/min. Fluorescent images of UCNPs and images of the UCNP/KNF composite films were captured using the built-in camera of Xiaomi CIVI 1S smartphone (Xiaomi Corporation, Beijing, China). The morphologies of composite film consisting of UCNPs and KNFs were examined using Phenom ProX scanning electron microscopy (SEM) (Phenom-World, Eindhoven, The Netherlands). Fourier transform infrared spectroscopy (FTIR) was performed using a TENSOR II Fourier transform infrared spectrometer (Bruker, Rheinstetten, Germany) equipped with an integrated platinum ATR accessory. Raman spectra were acquired using a DXRxi Raman imaging microscope (ThermoFisher Scientific Corporation, Waltham, MA, USA). UV–visible-IR absorption spectra were obtained using an ultraviolet–visible–near-infrared Cary5000 spectrophotometer (Agilent Technologies Inc., Santa Clara, CA, USA). 

### 2.3. CNPs Synthesi

Nanomaterials exhibiting diverse luminescent properties can be synthesized through the incorporation of various lanthanide ions [27]. UCNPs containing various rare-earth ions were synthesized following the procedures outlined in Li et al. [28]. The composition of materials in the core is detailed in Table S1. The shell material consisted of Y^3+^ as the host ion, with an additional layer of inert NaYF_4_ covering the outer surface of the core. The synthesis procedure comprised the dissolution of rare-earth chloride, the formation of crystal nuclei, and the growth of grains. The synthesized UCNPs were denoted as NaYF_4_: Er@NaYF_4_, NaErF_4_@NaYF_4_, and NaErF_4_: Tm@NaYF_4_ based on the distinct elements present in UCNPs. Utilizing NaYF_4_: Er (12.5 mol%) @NaYF_4_ particles as an illustration, the synthesis procedure is outlined as follows.

#### 2.3.1. Synthesis of NaYF_4_: Er UCNPs

Initially, a solution was prepared by adding 0.875 mmol of YCl_3_ and 0.125 mmol of ErCl_3_ to deionized water in a 100 mL three-necked flask. The mixture was vigorously stirred using a magnetic stirrer until a clear solution was obtained. Subsequently, 6 mL of OA and 15 mL of ODE were introduced into the solution, which was then heated to 100 °C for approximately 10 min to eliminate the water content until no bubbles were visible on the surface. Secondly, the mixture was heated to 156 °C and maintained at this temperature for 30 min. Subsequently, the resulting clear yellow solution was cooled to room temperature. Subsequently, a methanol (CH_3_OH) solution containing 2.5 mmol of NaOA (0.761 g) and 4 mmol of NH_4_F (0.1482 g) was added and maintained for 30 min at room temperature. After stirring the mixture at 70 °C for approximately 30 min and at 100 °C for 10 min to ensure complete removal of CH_3_OH and water, the reaction vessel was subsequently heated to 300 °C for 1 h, and then allowed to cool to room temperature. The entire procedure was conducted in the presence of a mild stream of argon gas. After being precipitated with 10 mL of acetone (C_3_H_6_O) and washed multiple times with C_6_H_12_ and C_3_H_6_O, UCNPs containing 12.5 mol% of Er^3+^ doping were precipitated and then redispersed in 6 mL of C_6_H_12_.

#### 2.3.2. Synthesis of NaYF_4_: Er@NaYF_4_ Core–Shell UCNPs

The aqueous solution containing 1 mmol of YCl_3_ was combined with 1 mL of water, followed by the addition of 6 mL of OA and 15 mL of ODE. The mixture was subjected to heating at 156 °C for a duration of 30 min. When the transparent yellow solution was cooled to 80 °C, the as-synthesized NaYF_4_: Er UCNPs, with an Er^3+^ doping concentration of 12.5 mol% in 6 mL of C_6_H_12_, were introduced as a nanocore. The reactant mixture was consistently cooled to ambient temperature. Subsequently, a CH_3_OH solution containing 2.5 mmol of NaOA (0.761 g) and 4 mmol of NH_4_F (0.1482 g) was added and maintained for 30 min at room temperature. After stirring the mixture at 70 °C for approximately 30 min and at 100 °C for 10 min to eliminate CH_3_OH and water, the reactant mixture was subsequently heated to 300 °C for 1 h and then allowed to cool to room temperature. The entire procedure was conducted under a gentle stream of argon gas. After precipitation with 10 mL of C_3_H_6_O and washing several times with C_6_H_12_ and C_3_H_6_O, NaYF_4_: Er@NaYF_4_ core–shell UCNPs were obtained, precipitated, and then redispersed in 6 mL of C_6_H_12_.

#### 2.3.3. PEG Modification of NaYF_4_: Er@NaYF_4_ Core–Shell UCNPs

**Scheme 1:**
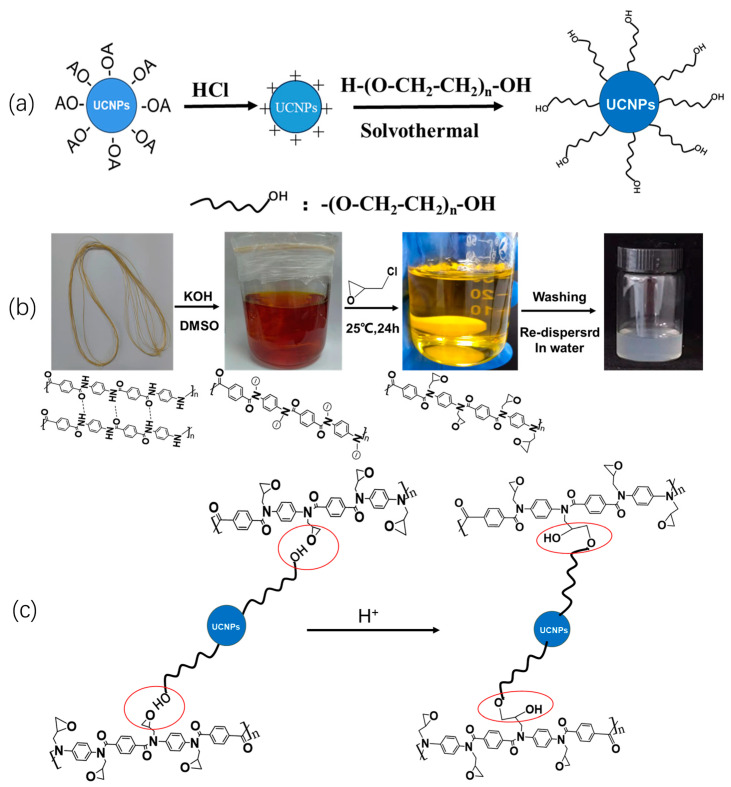
Multistage fabrication process: (**a**) PEG modification of UCNPs; (**b**) preparation of M-KNFs; (**c**) synthesis of UCNP/KNF composites.

A schematic illustration depicting the modification of UCNPs is presented in Scheme 1a. A 3 mL solution of UCNPs in C_6_H_12_ was precipitated with CH_3_CH_2_OH, followed by removal of the supernatant through centrifugation. The wet precipitate was then re-dispersed in 5 mL of 0.1M HCl, and subjected to ultrasonic treatment at 45 °C for 60 min. Following this step, the nanoparticle solution underwent centrifugation at 14,000 rpm for 30 min. Subsequently, the precipitation was washed twice with deionized water. Through this procedure, the ligand OA present on the surface of the nanoparticles was eliminated and substituted with H^+^ ions, resulting in the dispersion of ligand-free nanoparticles in 5 mL of deionized water.

PEG (50 mg) was dissolved in 45 mL of deionized water, followed by the gradual addition of a 5 mL solution of ligand-free UCNPs under stirring conditions. After stirring for 2 h, 50 mL of diethylene glycol (DEG) was added, and the mixture was heated continuously at 105 °C for 1 h to remove water. Subsequently, the mixture was transferred to a Teflon-lined autoclave and maintained at 105 °C for 1 h. Upon reaching room temperature, the suspension underwent centrifugation at 14,000 rpm for 30 min to obtain the wet precipitate. After undergoing multiple washes with CH_3_CH_2_OH and water, the negatively charged UCNPs modified with PEG were obtained and subsequently redispersed in 20 mL of deionized water. The solid content of the sample was quantified, and the concentration was set at 6 mg/mL. The specimen was refrigerated for future utilization.

### 2.4. Synthesis and Water-Soluble Modification of KNFs

KNFs were prepared using the method described by Kotov research group [21]. KNF/DMSO dispersion was subsequently epoxidized following the procedure outlined in Zhang et al. [29]. Nanofiber dispersion (100 mL, 2 mg/mL) was transferred into a 250 mL beaker, followed by the addition of 0.135 mL of ECH. The resulting mixture was continuously stirred for 24 h at room temperature. During the agitation process, a significant transition in the solution’s color can be observed, shifting gradually from a dark red hue to a golden yellow shade, accompanied by a corresponding reduction in the solution’s viscosity. Subsequently, DMSO solution containing ECH-modified Kevlar nanofibers (M-KNFs) was subjected to centrifugal washing at 12,000 rpm until reaching a neutral pH, resulting in the formation of M-KNF jelly. The solid content of M-KNFs was then determined. M-KNFs adhesive was subsequently dispersed in deionized water at a constant volume (3 mg/mL) with the assistance of ultrasonication. This dispersion exhibited long-term stability without stratification. The schematic illustration depicting the preparation of M-KNFs and accompanying images are presented in Scheme 1b.

### 2.5. Synthesis and Film Preparation of UCNP/KNF Composites

The synthesis of UCNPs/KNFs involves the ring-opening reaction of the -OH group on the PEG-modified UCNPs with the epoxide group on the Kevlar fiber. The synthesis mechanism is illustrated in Scheme 1c. A specific volume of epoxidized KNF aqueous solution including 30 mg of epoxidized KNFs was combined with PEG-modified aqueous solutions of various types of UCNPs: NaYF_4_: Er (12.5 mol%) @NaYF_4_, NaErF_4_@ NaYF_4_, and NaErF_4_: Tm (0.5 mol%) @NaYF_4_. The components were measured and placed in reagent bottles following the proportions outlined in Table S2. Subsequently, the mixtures were adjusted to a pH of about 4 by acetic acid and vigorously stirred for 12 h at room temperature. Subsequently, the wet precipitated film was acquired through vacuum filtration. After self-demolding or manual demolding, the composite self-supporting films of UCNPs/KNFs with 45 mm diameters were obtained.

## 3. Results and Discussion

### 3.1. Morphology and Spectroscopic Properties of UCNPs Modified with PEG

**Figure 1:**
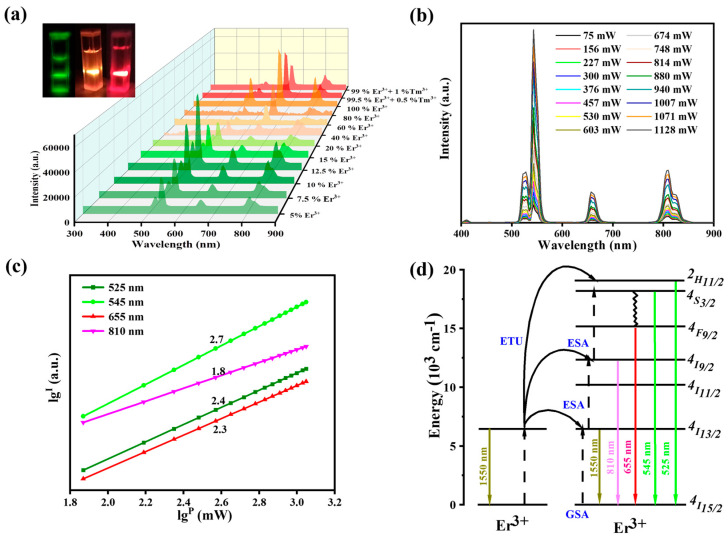
Luminescent properties of UCNPs under 1550 nm excitation: (**a**) PL spectra (2.5 W/cm^2^) including the pictures (green: NaYF_4_: Er^3+^ @NaYF_4_; yellow: NaErF_4_ @NaYF_4_; red: NaErF_4_: Tm^3+^ @NaYF_4_) in the set; (**b**) upconversion emission spectra of NaYF_4_:Er (12.5 mol%)@NaYF_4_ under different excitation powers; (**c**) emission intensity as a logarithmic function of excitation power for NaYF_4_:Er(12.5 mol%)@NaYF_4_ (the numbers next to the lines refer to the slope of each line); (**d**) schematic energy level diagram showing the upconversion process for UCNPs (GSA: ground state absorption; ESA: excited state absorption; ETU: energy transfer upconversion).

The luminescent characteristics of UCNPs exhibit variations based on the presence of different doping ions. In Figure 1a, all spectral lines exhibit prominent emission peaks at 525 nm, 545 nm, 655 nm, and 810 nm, which correspond to transitions between energy levels ^2^H_11/2_–^4^I_15/2_, ^4^S_3/2_–^4^I_15/2_, ^4^F_9/2_–^4^I_15/2_, and ^4^I_9/2_–^4^I_15/2_ [30]. In the case of NaYF_4_: Er (5–80 mol%) @NaYF_4_, the intensity of green light (525 nm and 540 nm) surpasses that of red light (655 nm) and near-infrared light (810 nm) when Er^3+^ content is low (5–12.5 mol%). As Er^3+^ content increases, the luminescent performance improves across all three bands. When the doping content of Er^3+^ reaches 12.5 mol%, the luminescent performance reaches its peak. Subsequently, the luminescent intensity exhibited a decline as the doping content increased, reaching its lowest point at approximately 40%. With the increase in Er^3+^ concentration, the luminous intensity of the red light band (655 nm) surpasses that of the green light (525 nm and 540 nm) and near-infrared light (810 nm). Additionally, the luminous intensity demonstrates a positive correlation with Er^3+^ content increment. When the doping content of Er^3+^ reached 100 mol%, the luminescent efficiency of NaErF_4_@NaYF_4_ increased, although it was lower compared to NaYF_4_: Er (12.5 mol%) @NaYF_4_. This phenomenon can be attributed to cross-relaxation between ions [31], which predominantly influences the shift in emission color from green to red and the near-infrared region in NaYF_4_: Er^3+^@NaYF_4_ UCNPs as the concentration of Er^3+^ ions increased from 12.5 mol% to 100 mol%. The research indicated that the introduction of a small quantity of Tm^3+^ in the specimen significantly reduced the intensity of green and near-infrared light in NaErF_4_@NaYF_4_. This decrease can be attributed to the role of Tm^3+^ as an energy trap, leading to a higher number of photons undergoing non-radiative transitions to a ^4^F_9/2_ excited state level, ultimately resulting in red light emission [32,33]. In summary, three aqueous nanoparticles, namely NaYF_4_: Er (12.5 mol%) @NaYF_4_, NaErF_4_ @NaYF_4_, and NaErF_4_: Tm (0.5 mol%) @NaYF_4_, were chosen for the experiment. The images of these UCNP suspensions when excited under 1550 nm are illustrated in the set.

Figure 1b displays the fluorescence spectrum of NaYF_4_: Er (12.5 mol%) @NaYF_4_ under varying laser excitation powers. It can be concluded that the luminous intensity increases with the rise in excitation power. According to the logarithmic relationship between the luminescent intensity and excitation power (Figure 1c) of NaYF_4_: Er(12.5 mol%) @NaYF_4_ irradiated by a laser with different powers at 1550 nm, the luminescence of green light (525 nm and 545 nm), red light (655 nm), and near-infrared light (810 nm) exhibits three-photon absorption, three-photon absorption, three-photon absorption, and two-photon absorption, respectively. The same conclusions apply to NaErF_4_ @NaYF_4_ and NaErF_4_: Tm (0.5 mol%) @NaYF_4_ (Figure S1). The results indicate that the luminescent intensity of upconversion is influenced by the addition of varying concentrations of rare-earth ions, while the upconversion mechanism remains consistent across all emission regions (Figure 1d).

**Figure 2:**
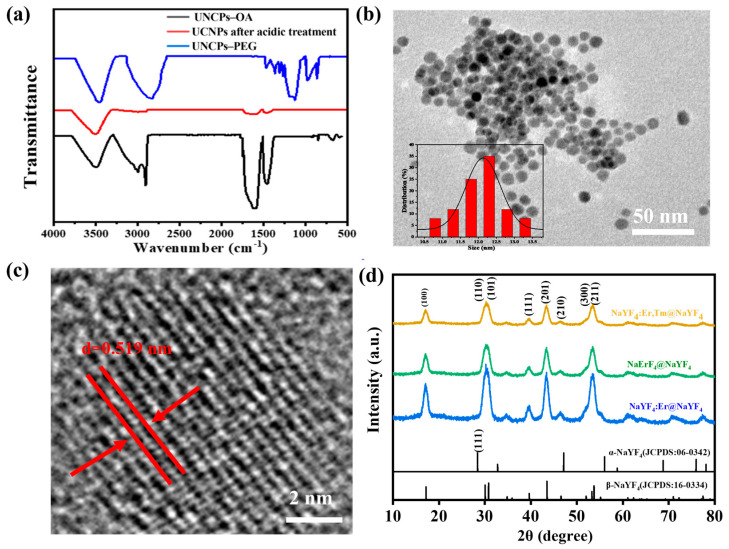
Characterizations of UCNPs: (**a**) FTIR spectra of UCNP–OA, UCNPs after acidic treatment, and UCNP–PEG; (**b**) TEM image for NaYF_4_: Er^3+^@NaYF_4_ nanoparticles together with the size distributions; (**c**) HRTEM image for NaYF_4_: Er^3+^@NaYF_4_ nanoparticles; (**d**) XRD patterns of different UCNPs.

In the FTIR spectra of UCNP–OA, the UCNPs post-acidic treatment, and UCNP–PEG (Figure 2a), the stretching vibration peak of -OH is observed between 3200 cm^−1^ and 3700 cm^−1^, while the symmetric and asymmetric stretching vibration peak of -CH_2_ is approximately 2900 cm^−1^. After PEG modification, it was observed that the peak intensity increased. The peak at 1638 cm^−1^ corresponded to the asymmetric stretching vibration of carbon dioxide (CO_2_), while the peak at 1563 cm^−1^ represented the asymmetric stretching vibration of carbon monoxide (CO). Additionally, peaks at 843 cm^−1^, 965 cm^−1^, and 1107 cm^−1^ were associated with the bending and stretching vibrations of -COC-. The results suggest that OA ligands were removed and PEG ligands were bound to the nanoparticles following acid treatment.

TEM and high-resolution transmission electron microscopy (HRTEM) images of NaYF_4_:12.5% Er@NaYF_4_ are presented in Figure 2b,c. The analysis indicates that the suspension exhibits good dispersibility in water and shows a relatively uniform size distribution, with an average particle size of approximately 12 nm. The diffraction lattice displays prominent stripes when observed in the high-resolution image. The lattice spacing measures 0.519 nm, indicating the presence of the (001) crystal plane within the hexagonal crystal structure. We can infer that the conclusion also applies to both NaErF_4_@NaYF_4_ and NaErF_4_: 0.5%Tm @NaYF_4_ UCNPs because they were synthesized by the same method.

XRD tests were conducted on PEG-coated NaYF_4_:12.5% Er@NaYF_4_, NaErF_4_@NaYF_4_, and NaErF_4_: 0.5%Tm @NaYF_4_ to investigate the crystal structures of various UCNPs (Figure 2d). XRD patterns of the three UCNPs exhibited diffraction peaks at 2θ = 16.9°, 30.2°, 39.4°, 43.3°, and 53.4°, respectively. This pattern aligns with the hexagonal NaYF_4_ standard card, with the absence of any additional diffraction peaks. Therefore, the three nanomaterials exhibit a simple hexagonal crystal structure.

### 3.2. KNF Morphology

**Figure 3:**
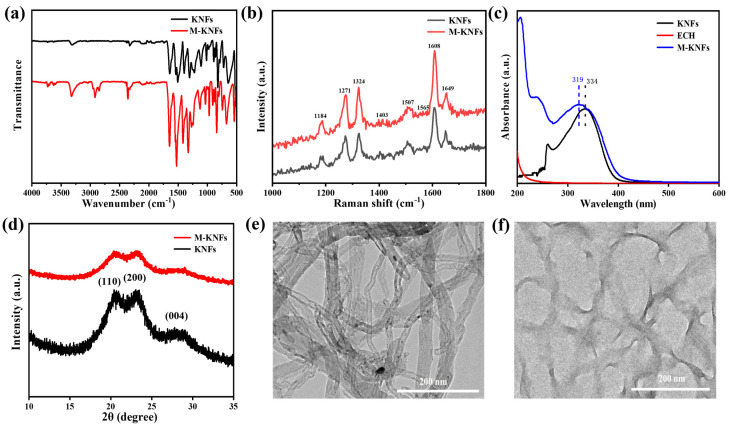
Comprehensive characterization of KNFs and M-KNFs: (**a**) FTIR spectra; (**b**) Raman spectra; (**c**) UV–vis absorbance spectra; (**d**) XRD patterns; (**e**) TEM images of KNFs; (**f**) TEM images of M-KNFs.

The FTIR (Figure 3a) reveals distinctive peaks associated with specific functional groups. The peak observed at 3318 cm^−1^ corresponds to the stretching vibration of -NH, while the peak at 1652 cm^−1^ signifies the stretching vibration of C=O. Additionally, the deformation vibration peak at 1537 cm^−1^ is indicative of N-H deformation, the peak at 1519 cm^−1^ represents the stretching vibration of C=C, and the peak at 1306 cm^−1^ is attributed to the stretching vibration of C-N within the ring structure [34,35]. These spectral features are characteristic infrared peaks of KNFs. Upon comparison with the FTIR of M-KNFs obtained through ECH modification, it was observed that the peaks at 1537 cm^−1^ were no longer present. Instead, stretching vibration peaks of epoxy groups emerged at 956 cm^−1^. Furthermore, the peaks at 2915 cm^−1^ and 2850 cm^−1^ were identified as -CH_2_ and -CH stretching vibrations, suggesting the successful grafting of epoxypl onto KNFs.

Additionally, the chemical composition and molecular structure of KNFs and M-KNFs were analyzed using Raman spectroscopy (Figure 3b). The Raman spectra of KNFs exhibit several characteristic peaks, each corresponding to different vibration modes of the material. Based on the characterization results in Zhang et al. [29], the spectral peaks observed at 1184 cm^−1^, 1271 cm^−1^, 1324 cm^−1^, 1507 cm^−1^, and 1608 cm^−1^ are associated with C-C vibrations within the benzene ring. Additionally, the spectral peak at 1403 cm^−1^ is linked to the C-H bending vibration within the benzene ring. The peak observed at 1565 cm^−1^ is associated with the N-H bending vibration and C-N stretching vibration of the amide bond, while the peak at 1649 cm^−1^ is linked to the C=O stretching vibration of the amide bond. The Raman spectra of M-KNFs closely align with the peaks of KNFs, suggesting that the alteration of epoxy groups has minimal impact on the molecular chain structure.

To validate the presence of epoxy groups, the UV–vis absorption spectra of KNFs and M-KNFs were analyzed (Figure 3c). The UV absorption spectra of KNFs exhibit an absorption peak at 334 nm, indicative of the π-π* transition of C-C bonds within the aromatic ring structure of KNFs. The ultraviolet absorption peak of M-KNFs exhibits a blue shift, with the spectral peak occurring at 319 nm. The attachment of epoxypropyl to KNF molecules results in a decrease in the π electron content within the molecular chains of KNFs [36]. The results indicate the successful addition of the epoxy-propyl group.

To advance the investigation of the microstructure of KNFs and M-KNFs, the samples underwent XRD analysis (Figure 3d). The spectral peaks observed at approximately 20.5°, 23.2°, and 28.6° are attributed to the characteristic diffraction peaks of KNFs, corresponding to the crystal faces (110), (200), and (004), respectively [29]. The XRD pattern of M-KNFs exhibits identical diffraction peak positions to KNFs, albeit with a reduction in the intensity of certain peaks. This alteration could be attributed to the successful incorporation of epoxy groups, leading to a modification in the molecular chain arrangement.

Transmission electron microscopy (TEM) images depicting KNF and M-KNF solutions are presented in Figure 3e,f. KNFs exhibit excellent dispersion within the solution, showcasing a 3D network structure, numerous branches, and a pristine fiber surface. M-KNFs exhibit a substantial particle size and form a 3D network structure in solution, demonstrating a significant level of adhesion. The phenomenon could be attributed to the incomplete epoxidation reaction of KNFs, leading to partial hydrolysis and the formation of hydrogen bonds within the solution.

### 3.3. Morphology, Spectroscopic Properties, and Performance Analysis of UCNP/KNF Composite Films

**Figure 4:**
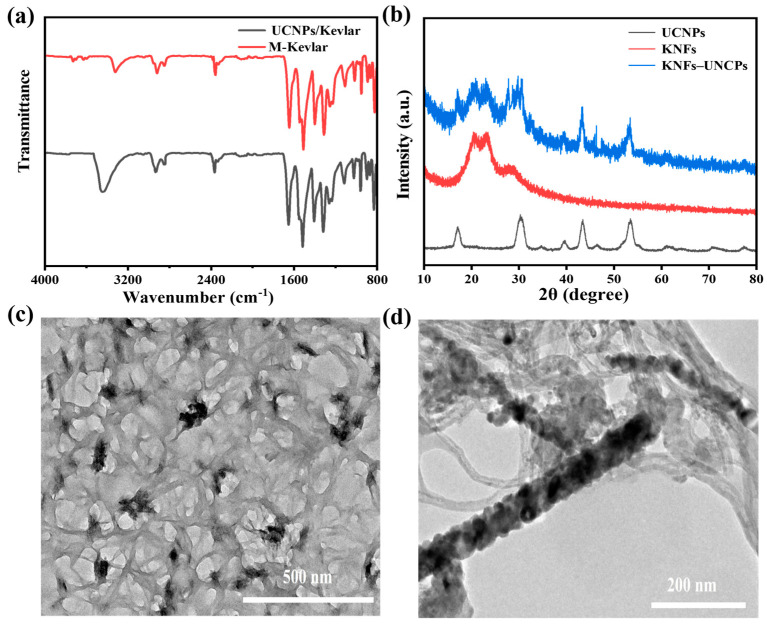
Analytical characterization of UCNP/KNF composite: (**a**) FTIR spectra; (**b**) XRD pattern; (**c**,**d**) TEM images of UCNPs/KNFs.

Figure 4a illustrates the FTIR of KNFs and UCNPs/KNFs. Following the integration of KNFs and UCNPs (NaYF_4_:Er^3+^@NaYF_4_), a -OH stretching vibration peak emerged at approximately 3439 cm^−1^, overlapping with the -NH stretching vibration peak near M-KNFs at 3327 cm^−1^. Furthermore, the intensities of the -CH_2_ and -CH stretching vibration peaks at 2915 cm^−1^ and 2850 cm^−1^, respectively, exhibited an increase, suggesting an augmentation in the presence of -CH_2_ and -CH groups within the material.

The XRD pattern of the composite film containing UCNPs and KNFs is illustrated in Figure 4b. Diffraction peaks of the composite film are observed at 2θ = 16.9°, 30.2°, 39.4°, 43.3°, and 53.4°, which correspond to the standard hexagonal phase crystal faces of UCNPs, specifically (100), (101), (111), (201), and (211), respectively. The peaks observed in the composite film at 2θ = 20.5°, 23.2°, and 28.6° are associated with the crystal faces (110), (200), and (004) of M-KNFs. The diffraction peaks observed in the composite films exhibit diffraction patterns characteristic of both the pure phase UCNPs and Kevlar, with their positions remaining largely unaltered. The results show the successful synthesis of composite films of UCNPs/KNFs. Figure 4c,d shows TEM images of UCNPs/KNFs at various magnifications. The TEM analysis revealed that NaYF_4_:Er^3+^@NaYF_4_ UCNPs were slightly aggregated but evenly distributed within the 3D network structure of KNFs. In Figure 4d, a uniform layer of UCNPs can be observed to grow on the surface of certain fibers, suggesting that nanoparticles are well bonded with KNFs. The modification is considered stable due to the effective trapping of UCNPs by the micrometric KNFs. Even if certain UCNPs aggregate, the micrometric size of the fiber allows for the incorporation of UCNPs without compromising the integrity of the final product.

**Figure 5:**
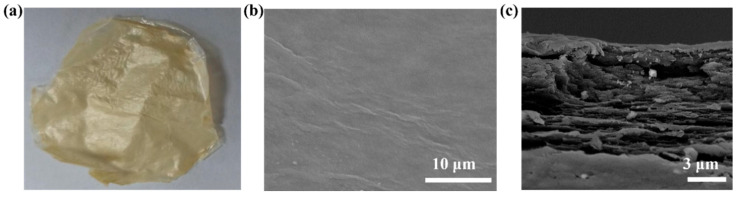
Characterizations of UCNP/KNF composite film: (**a**) digital picture; (**b**,**c**) surface and cross-section views of SEM images.

Self-supporting films were produced through vacuum-assisted filtration and subsequent drying. In the digital photograph (Figure 5a), the self-supporting composite film prepared is light yellow. The surface microstructure of the composite film appears flat and wrinkled (Figure 5b), a characteristic attributed to the shrinkage of KNFs during the drying process. The composite film (Figure 5c) has a thickness of approximately 10 microns and a fiber layer stack shape that distributes UCNPs.

**Figure 6:**
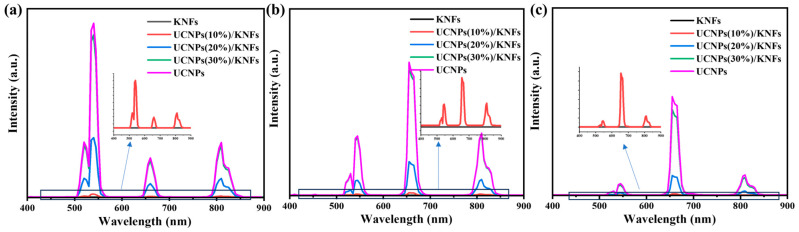
PL spectra of KNFs, UCNPs/KNFs, and UCNPs (UCNPs: NaYF_4_: 12.5%Er @NaYF_4_ (**a**), NaErF_4_ @NaYF_4_ (**b**) and NaErF_4_: 0.5%Tm @NaYF_4_ (**c**)).

Figure 6 shows the PL spectra of the KNF, UCNP/KNF, and UCNP aqueous solutions evaluated at 1550 nm, respectively. We ensure the same quality of KNFs in the KNF solution and UCNP/KNF solution, and the same quality of UCNPs in the UCNP solution and UCNP (30%)/KNF solution. Upon excitation with the 1550 nm laser, the emission spectra of UCNPs/KNFs all exhibited three distinct upconversion emission bands at 525, 545, and 655 nm, just like UCNPs, and as expected, no luminescence was detected for the KNFs. It is also revealed that the relative emission intensity of UCNP/KNF composite solutions increases with increasing amounts of UCNPs used. Then, we can infer, in a qualitative way, that no quenching effects were detected by increasing the mass content of UCNPs from 10% to 30%, as the experimental conditions, such as excitation power and detection slits, remained constant throughout the measurement process. In this case, the UCNP (30%)/KNF suspensions (containing different UCNPs of NaYF_4_: 12.5%Er @NaYF_4_, NaErF_4_ @NaYF_4_, and NaErF_4_: 0.5%Tm @NaYF_4_, respectively) were used in the following vacuum filtration membrane process.

**Figure 7:**
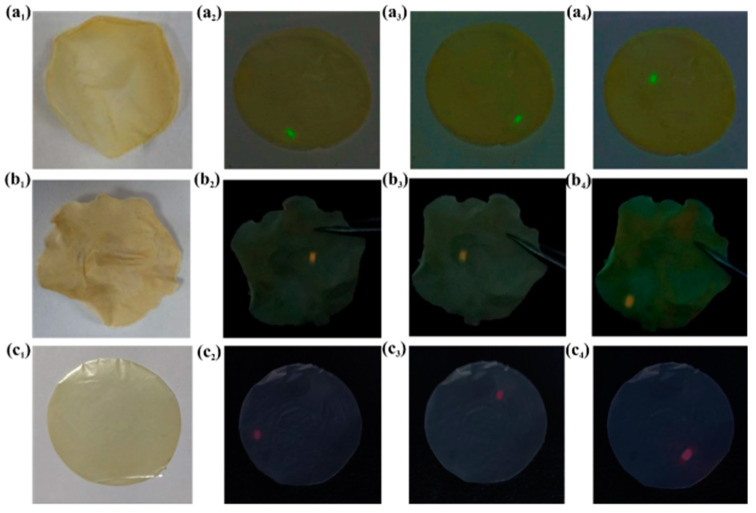
Visual comparison of UCNP/KNF composite films under different lighting conditions: (**a_1_**–**a_4_**) digital pictures of NaYF_4_: 12.5%Er @NaYF_4_/KNF film; (**b_1_**–**b_4_**) NaErF_4_ @NaYF_4_/KNF film; (**c_1_**–**c_4_**) NaErF_4_: 0.5%Tm @NaYF_4_/KNF film in natural light and in dark irradiated by 1550 nm.

Figure 7(a_1_,b_1_,c_1_) display the digital pictures of self-supported UCNP/KNF composite films fabricated by incorporating 30 wt.% of NaYF_4_: 12.5%Er @NaYF_4_, NaErF_4_ @NaYF_4_, and NaErF_4_: 0.5%Tm @NaYF_4_, respectively, under ambient lighting conditions. The composite films in the low-light environment underwent irradiation using a 1550 nm laser (12.5 W/cm^2^) at various positions, as illustrated in Figure 7(a_2_–a_4_,b_2_–b_4_,c_2_–c_4_). Distinct luminescence can be observed at various locations on the film when exposed to laser irradiation. The results indicate that UCNPs are evenly distributed within the composite film, leading to satisfactory luminescent performance of the composite film. Consequently, this material is anticipated to be utilized in the field of exhibition.

**Figure 8:**
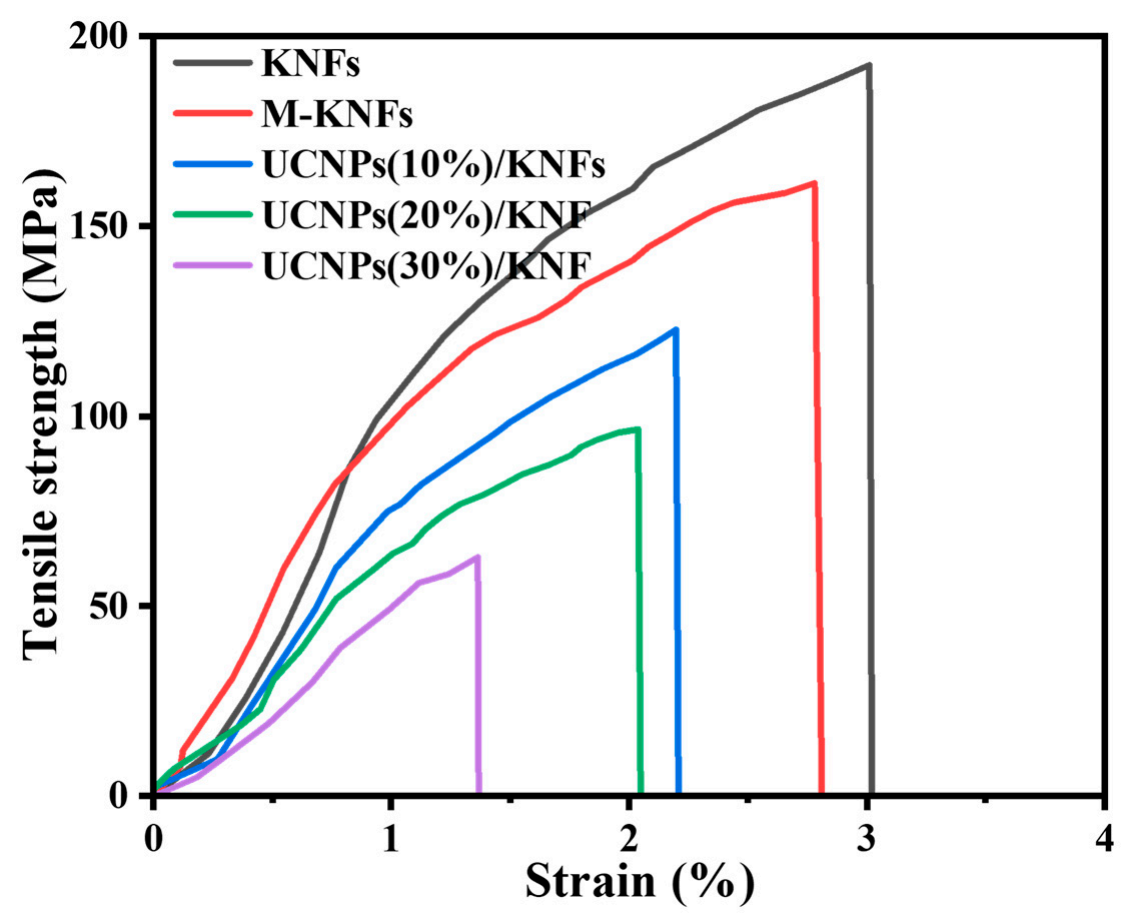
Comparative tensile stress–strain curves of pure KNFs, pure M-KNFs, and UCNP/KNF composite films with different contents of UCNPs.

Tensile tests were conducted on the composite films containing varying amounts of UCNPs. Figure 8 illustrates the tensile stress–strain curves of KNF, M-KNF, and UCNP/KNF composite films containing varying amounts of NaErF_4_: 0.5%Tm @NaYF_4_. KNFs exhibit significant tensile strength, with a Young’s modulus reaching 6.39 GPa (Figure 8 and Table S3). M-KNFs exhibit a slightly reduced strength, with a Young’s modulus of 5.80 GPa. Subsequently, as the number of UCNPs increased, the Young’s modulus decreased progressively. The presence of doped UCNPs influences the dense arrangement of long-chain fibers, leading to a reduction in packing density. However, the altered KNFs remain stable and appropriate for additional utilization. In the drawing process, the force needed for pulling decreases as the number of UCNPs rises.

The electron population of lanthanide rare-earth ions at the thermally coupled energy level follows the Boltzmann distribution. The relationship between temperature and the integral intensity ratio of the upconversion emission peak of two thermally coupled energy levels is described by the following equation [37,38]:

FIR = I_U_/I_L_ = C × exp[−ΔE/(kT)]. (1)

where I_U_ and I_L_ are the integral intensity of the emission peaks of the upper and lower levels of the two thermally coupled levels, respectively; C is a constant related to the properties of rare-earth luminescent ions themselves; ΔE is the energy level difference between the two thermally coupled levels; k is the Boltzmann constant; and T is the absolute temperature of the sample to be measured. If the rare-earth ions doped in the substrate material are specified, with fixed constants C and ΔE, the fractional intensity ratio (FIR) value becomes solely dependent on the absolute temperature of the sample. The temperature of the measured sample can be indirectly determined by analyzing the ratio of the luminescent intensity between the two thermal coupling levels of the system. The study also establishes a correlation between the two peak integral intensity ratios at 525 nm and 545 nm and the absolute temperature of the emission spectrum of Er^3+^ luminous ions in UCNPs.

To enhance the precision of the research results, a composite film containing 30% NaYF_4_: Er (12.5 mol%) @NaYF_4_ and exhibiting relatively high luminescent performance was chosen for experimentation. To assess the temperature-sensing performance of the material under 1550 nm laser irradiation, it is essential to measure the variable temperature upconversion spectra of the composite. Ensuring the sample remains unaffected by thermal influences during the test is crucial. Consequently, the upconversion spectrogram of the sample underwent initial testing within a 30 min period of uninterrupted irradiation using a 2.5 W/cm^2^ 1550 nm laser (Figure S2a). The normalized spectral peak and integrated emission peak intensity were utilized to analyze the temporal evolution of the ratio between two green light peaks (Figure S2b). The study revealed that the ratio between two peaks of green light remained constant over a period of 30 min. The results indicate that the thermal impact of an excitation density of 2.5 W/cm^2^ on the material during the test does not influence the subsequent temperature-change test.

**Figure 9:**
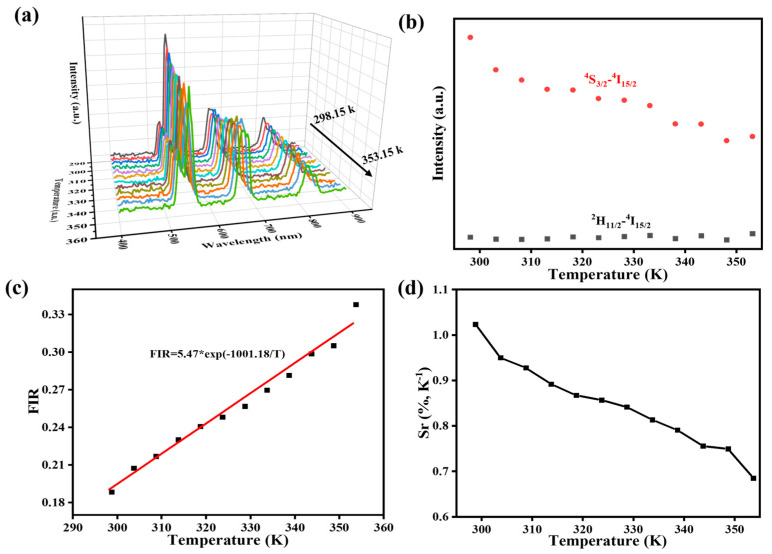
Temperature-dependent luminescence characterization of UCNPs/KNFs: (**a**) upconversion spectra of UCNP/KNF films upon 1550 nm (2.5 W/cm^2^) at different temperatures; (**b**) dependence of the integrated intensities of ^4^S_3/2_–^4^I_15/2_ and ^2^H_11/2_–^4^I_15/2_ emissions on temperature; (**c**) temperature-dependent FIR of UCNPs/KNFs; (**d**) temperature sensitivity of UCNPs/KNFs as a function of temperature.

Based on this premise, the upconversion spectra of UCNPs/KNFs were examined within the temperature range of 298 K to 353 K using a constant laser excitation power density of 2.5 W/cm^2^ (Figure 9a). Subsequently, the data were normalized, and the peak corresponding to the bright green light was determined by integrating the area. The luminescent intensity of the two coupled levels exhibits distinct trends as the temperature increases (Figure 9b). The transition from ^4^S_3/2_ to ^4^I_15/2_ (545 nm) exhibited a consistent decrease as the temperature rose, whereas the transition from ^2^H_11/2_ to ^4^I_15/2_ (525 nm) displayed a trend of dynamic equilibrium. Consequently, the ratio is inclined to rise as the temperature increases. The temperature-sensing curve of the sample is derived through linear fitting, by employing the fitting equation that describes the relationship between the integral intensity ratio and temperature (Figure 9c). The fitting equation is expressed as follows:

FIR = 5.47 × exp(−1001.18/T).(2)

The calculation indicates that ΔE/k is 1001.18 and ΔE is 696.4 cm^−1^, which closely approximates the actual energy difference between two green light levels, 700.3 cm^−1^. This suggests that the fitting results are precise. The computed results in this investigation closely align with the data presented in comparable studies (Table 1), thereby providing additional validation for the precision of the temperature-sensing curve established in this study.

Additionally, in the practical implementation of temperature sensors, it is crucial to pay attention to significant indicators. The equation illustrating the correlation between the sensitivity of sample temperature sensing and temperature is given as follows:

S_r_ = (1/FIR) × [d(FIR)/dT] × 100% = ΔE/(kT^2^). (3)

The temperature-sensing sensitivity curve (Figure 9d) of the UCNP/KNF composite sample is obtained by incorporating the C value and ΔE/k into Equation (3). The sensitivity varies from 0.0068 to 0.0102.

## 4. Conclusions

This study synthesized a series of UCNPs emitting various upconversion light levels, and their morphology and spectral performance were analyzed. The product exhibited green, yellow, and red upconversion when subjected to laser excitation at 1550 nm. Upon the addition of 12.5 mol% Er^3+^ content, the UCNPs exhibit superior luminescent performance at green light wavelengths (525 nm and 540 nm) compared to red light (655 nm) and near-infrared light (810 nm). This study elucidates the primary mechanism of upconversion luminescence in UCNPs when excited by 1550 nm near-infrared light.

The synthesized NaYF_4_: Er (12.5 mol%) @NaYF_4_, NaErF_4_ @NaYF_4_, and NaErF_4_: Tm (0.5 mol%) @NaYF_4_ were effectively integrated into KNFs. The Kevlar samples were analyzed to investigate their spectroscopic characteristics, demonstrating the exceptional qualities of Kevlar as a matrix for luminescent UCNPs. This paper presents, for the first time, the optical and temperature-sensing performance of Kevlar-based UCNPs embedded in KNFs. The correlation between the luminescent intensity ratio of ^4^S_3/2–_^4^I_15/2_ (545 nm) and ^2^H_11/2_–^4^I_15/2_ (525 nm) emission bands with temperature suggests favorable sensing capabilities of the composite material when subjected to excitation. The properties of the synthesized composite allow for their application as luminescent markers for anti-counterfeiting purposes and as optical temperature sensors.

## Figures and Tables

**Table 1:** Values of ΔE/k for Ln^3+^.

Sample	ΔE/k(K^−1^)	Ref.
UCNPs/KNFs	1001.18	This work
NaYF_4_: Yb^3+^/Er^3+^	1028.5	[39]
NaLuF_4_: Yb, Er, Li	1102	[40]
NaYF_4_: Yb^3+^/Er^3+^	1044.16	[41]
NaYF_4_: Yb^3+^/Er^3+^	1082.10	[42]
NaGdF_4_: Yb^3+^/Er^3+^	1013.00	[43]

## Data Availability

Available upon request.

## Supplementary Materials

The following supporting information can be downloaded at: https://www.mdpi.com/article/10.3390/nano14090740/s1.
