# Supplementary material for: Luminescent/Temperature-Sensing Properties of Multifunctional Rare-Earth Upconversion Kevlar Nanofiber Composite under 1550 nm
Source: Nanomaterials (Basel). 2024 Apr 24;14(9):740. doi: 10.3390/nano14090740 (PMC11085643; doi:10.3390/nano14090740)
Supplement: Supplementary file 1 [file nanomaterials-14-00740-s001.zip › nanomaterials-2952411-supplementary.pdf]

Suppotring Information

Luminescence/Temperature-sensing properties of multifunctional rare-earth upconversion Kevlar nanofiber composite under 1550 nm

Juan Li[1], Shengang Xu[1,3], Yingliang Liu[1,3*], Shaokui Cao[1,2*]

[a]School of Materials Science and Engineering, Zhengzhou University, Zhengzhou 450001, People's Republic of China.
[b]Huanghe Science and Technology College, Zhengzhou, Henan 450063, People's Republic of China.
[c]Henan Key Laboratory of Advanced Nylon Materials and Application, Zhengzhou University, Zhengzhou 450001, People's Republic of China.
Correspondence: liuylxn@zzu.edu.cn (Y.L.); caoshaokui@zzu.edu.cn (S.C.)

Table S1 Chemical composition of Ln^{3+} in UCNPs

Y^{3+} content (mol%)	Er^{3+} content (mol%)	Tm^{3+} content (mol%)
95	5	0
92.5	7.5	0
90	10	0
87.5	12.5	0
85	15	0
80	20	0
60	40	0
40	60	0
20	80	0
0	100	0
1%	99.5	0.5
0	99	1

Table S2 UCNPs/KNFs composites with different mass ratio between UCNPs and KNFs

Sample number	$V_{M\text{-}KNFs}$ (mL) (3 mg/mL）	V_1 (mL) (6 mg/mL）	V_2 (mL) (6 mg/mL）	V_3 (mL) (6 mg/mL）	Deionized water (mL)
1	10	0	0	0	5
2-1	10	0.55	0	0	4.45
2-2	10	1.25	0	0	3.75
2-3	10	2.14	0	0	2.86
2-4	0	2.14	0	0	12.86
3-1	10	0	0.55	0	4.45
3-2	10	0	1.25	0	3.75
3-3	10	0	2.14	0	2.86
3-4	0	0	2.14	0	12.86
4-1	10	0	0	0.55	4.45
4-2	10	0	0	1.25	3.75
4-3	10	0	0	2.14	2.86
4-4	0	0	0	2.14	12.86

Note: V_1, V_2 and V_3 were volume of PEG-modified $NaYF_4$: Er(12.5 mol%) @$NaYF_4$, $NaErF_4$ @$NaYF_4$ and $NaErF_4$: Tm(0.5 mol%) @$NaYF_4$ aqueous solutions, respectively.

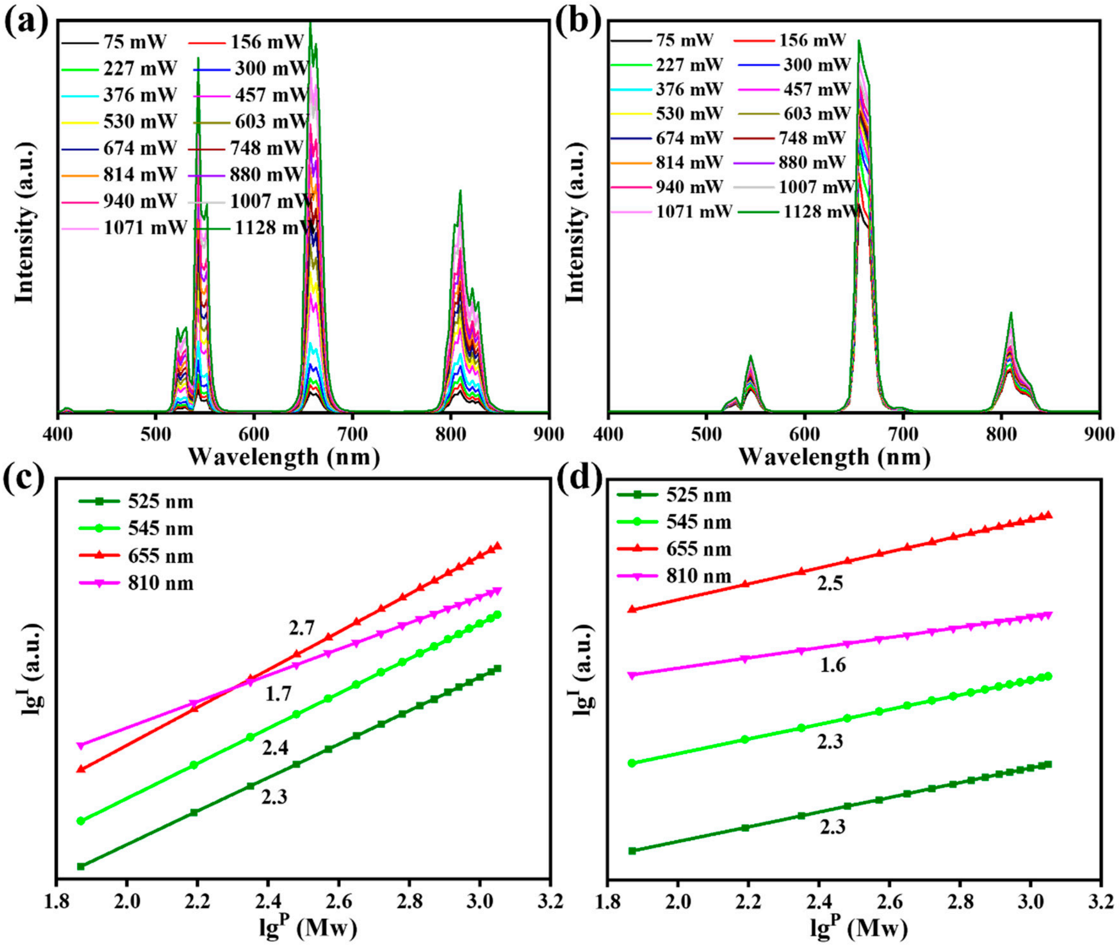

Figure. S1 Upconversion emission spectra of $NaErF_4$ @$NaYF_4$ (a) and $NaErF_4$: Tm(0.5 mol%) @$NaYF_4$ (b) under different excitation power at 1550 nm; emission intensity as a logarithmic function of excitation power for $NaErF_4$ @$NaYF_4$ (c) and $NaErF_4$: Tm(0.5 mol%) @$NaYF_4$ (d). The numbers next to the lines refers to the slope of each line.

Table S3 Mechanical tensile parameters of pure KNFs, pure M-KNFs, and UCNPs/KNFs composite films.

Sample	Tensile strength (MPa)	Strain at fracture (%)	Young's modulus (GPa)
Pure KNFs	192.37	3.01	6.39
Pure M-KNFs	161.23	2.78	5.80
UCNPs (10%)/KNFs	122.70	2.20	5.58
UCNPs (20%)/KNFs	96.60	2.04	4.73
UCNPs (30%)/KNFs	62.82	1.36	4.62

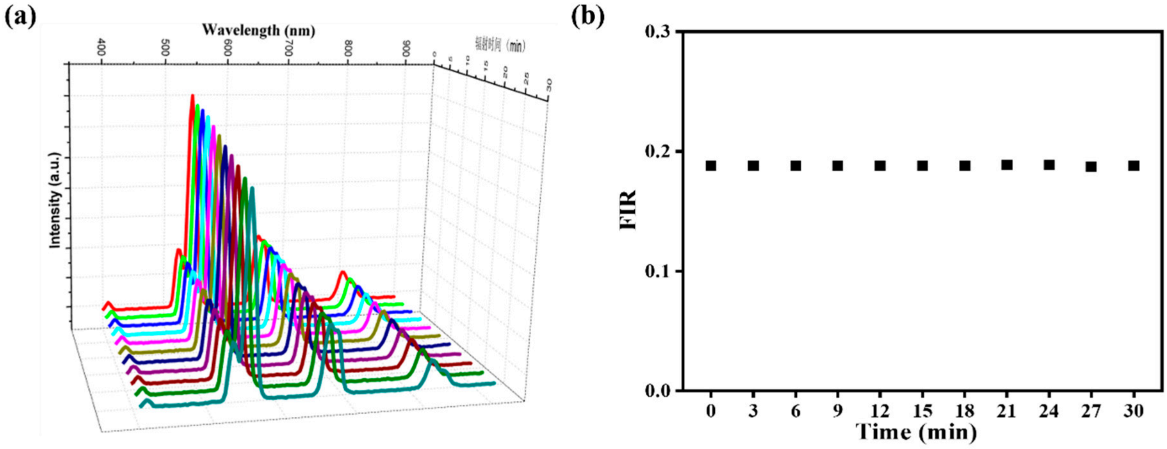

Figure. S2 The upconversion spectra of UCNPs/Kevlar films upon 1550 nm (2.5 W/cm^2) at different time (a); The dependence of FIR on irradiation time (b)
